# Longitudinal investigations of development in handball and its association with relative age effects

**DOI:** 10.3389/fspor.2025.1528684

**Published:** 2025-03-18

**Authors:** Jörg Schorer, Irene Faber, Dirk Büsch, Joseph Baker, Nick Wattie

**Affiliations:** ^1^ Institute of Sport Science, Carl von Ossietzky University Oldenburg, Oldenburg, Germany; ^2^ Tanenbaum Institute for Science in Sport, Faculty of Kinesiology and Physical Education, University of Toronto, Toronto, ON, Canada; ^3^ Faculty of Health Sciences, Ontario Tech University, Oshawa, ON, Canada

**Keywords:** talent, within-year effect, between-year effect, sport, bias

## Abstract

In this paper, we describe two studies on the association among long-term developmental outcomes and relative age effects. To extend or compliment the cross-sectional work done previously, these studies take different approaches to investigate the association of relative age effects on long-term development. In the first, a retrospective approach is taken, while in the second, developmental data for players over a 4-year period is considered. In study 1 the association between relative age effects and later performance at the adult level is presented. The results show different patterns for females and males. In the second study, development during the national youth development system in handball, over four points in time, are presented. Again, changes over time in birth quartile distribution can be seen. These studies suggest relative age researchers should embrace longitudinal designs. These types of approaches would allow explorations of the association of other variables with the observed relative age effects.

## 1 Introduction

In countries like Germany, where many sports set a cut-off date of January 1st, individuals born on December 31st are the youngest in their year group; a child born the next day (i.e., January 1st) is the oldest in the subsequent year group. Wattie et al.'s (1) model of the mechanisms behind “relative age effects” (RAEs) in sport posits that athletes born closer to the cut-off date used in their sport for age-group selection get developmental advantages due to their relative age compared to younger peers within the same cohort. Such advantages can manifest in increased opportunities for training, competition, and coaching attention (2). Despite the developmental nature of RAEs (3, 4), most research in this area has adopted cross-sectional or quasi-longitudinal approaches (3).

Lately, however, the developmental nature of the phenomena has begun to receive some attention (5). A study by Faber et al. (6), for example, showed that performance trajectories of the top 100 French table tennis players were associated with relative age. To the best of our knowledge no other study has taken a longitudinal approach to investigate these effects.

In this paper, we describe two studies on the association among long-term developmental outcomes and relative age effects. To extend or compliment the cross-sectional work done previously, these studies take different approaches to investigate the association of relative age effects on long-term development. In the first, a retrospective approach is taken, while in the second, developmental data for players over a 4-year period is considered.

## 2 Study 1—relationships between long-term success of handball and relative age

Previous studies on talent selection camps in German handball have shown classical relative age effects, with an over-representation of athletes born in quartile 1 (7–9). These studies have also shown that relative age effects are smaller in female athletes than in males (7–9). On the surface, these effects appear to decrease or even dimmish over time (e.g., when athletes reach adulthood) (10). However, to the best of our knowledge, no one has tested whether players from these talent camps who make it to the elite levels show the same distributions. Therefore, our primary aim in this study was to test whether this group of “early talents” who made it to the higher levels of performance demonstrated a relative age effect. In general, we expected a decrease in effect with age (3, 4) based on previous studies of handball talents (7–9).

A second aim was to identify players from the same birth years who made it to these higher levels, but were not considered for development at the lower level (i.e., were not participants in junior development programs). No previous studies have explored this type of comparison group, and therefore our analyses here were largely exploratory. For instance, it is possible that if talent is equally distributed over the year, and players from the talent camp are more likely to demonstrate a positive effect, the remaining players could demonstrate a reverse relative age effect with an over-representation in the fourth quartile. This relationship would suggest these “late bloomers” developed later in their career and were still able to make it to the top levels. A third aim of this study involved comparing the birth quartile distributions of early talents with late bloomers.

## 3 Study 1—methods

### 3.1 Sample

Birthdates for two groups were collected. First, the German Handball Federation provided the birthdates of participants involved in “talent selections” in 2010 and 2011. In those selections approximately 240 male and 240 female athletes participated each year (11). The players participating at these national talent camps had been previously selected by the twenty regional coaches. They were most often selected by the coaches' eye (12). The selection camps in 2010 and 2011 lasted for 5 days and included a range of tests as well as varying games, the athletes play. During these days national coaches selected players to form the basis for the youth national team.

We then determined league status for these members of this year group for the 2017/2018 season. The second group consisted of players from the same birth years, playing in the first three leagues in the 2017/2018 season, but who did not participate in those talent selection programs. Those players and their birthdates were retrieved from the various official websites of the German female and male handball leagues (http://www.hbl.de, http://www.hbfw.de, http://www.dhb.de/de/wettbewerbe/3–liga/uebersicht/) as well as from official websites (e.g., http://www. handball-world.de/news). From these sources, 108 males and 36 female players were identified.

### 3.2 Statistical analyses

To test RAEs in this study, birthdates were collected for all male players born from 1994 to 1995 (*n* = 542) and for all females born from 1995 to 1996 (*n* = 473) who either participated in their respective talent selections in 2010 (males with birth year 1994 and female birth year 1995) and 2011 (males with birth year 1995 and female birth year 1996) by the Germany handball federation (junior level) or played at the point of data collection in the highest three leagues (senior level).

At the selection camps, 449 male talents and 444 female talents participated (cf. Table 1). Of these junior participants, 119 male and 110 female players eventually ended up playing in the first to third leagues in German handball during the 2017/2018 season. Additionally, players with the same birth years who were in the first three leagues were identified (*n* = 93 male and 29 female players).

**Table 1:** Distribution of players at different career points for study 1.

Groups	Male	Female
At talent selection camp (and found in first three leagues in 2017/18 = early talents)	449	444
At talent selection camp and found in first three leagues in 2017/18 = early talents	119	110
NOT at talent selection camp and found in first three leagues in 2017/18 = late bloomers	93	29
Total numbers from both categories	542	473

Given that the cut-off date in handball is the first of January, players' birth months were re-coded to reflect his or her birth quartile (January–March = quartile 1, April–June = quartile 2, July–September = quartile 3 and October–December = quartile 4). Because previous research examining the birthdate distribution in Germany has shown roughly equal distributions across the quarters of the year (8), statistical analyses were conducted against this distribution. Chi-square tests were calculated over all leagues, for both sexes. Additionally, we separated “early talents” (i.e., current elite players who were selected as part of the early talent selection process) and “late bloomers” (i.e., current elite players who were not part of the early program). Comparisons of between year groups were not necessary for this sample, because they are only later combined to a 2-year group team.

All analyses were conducted with SPSS 29.0. The alpha-level was set to .05. Effect sizes (Cohen's w) were determined using G*power (13). For each effect size, the 90% confidence interval was calculated based on the noncentral Chi-square files provided online by Michael Smithson (http://www.michaelsmithson.online/stats/CIstuff/CI.html).^1^

## 4 Study 1—results

**Figure 1:**
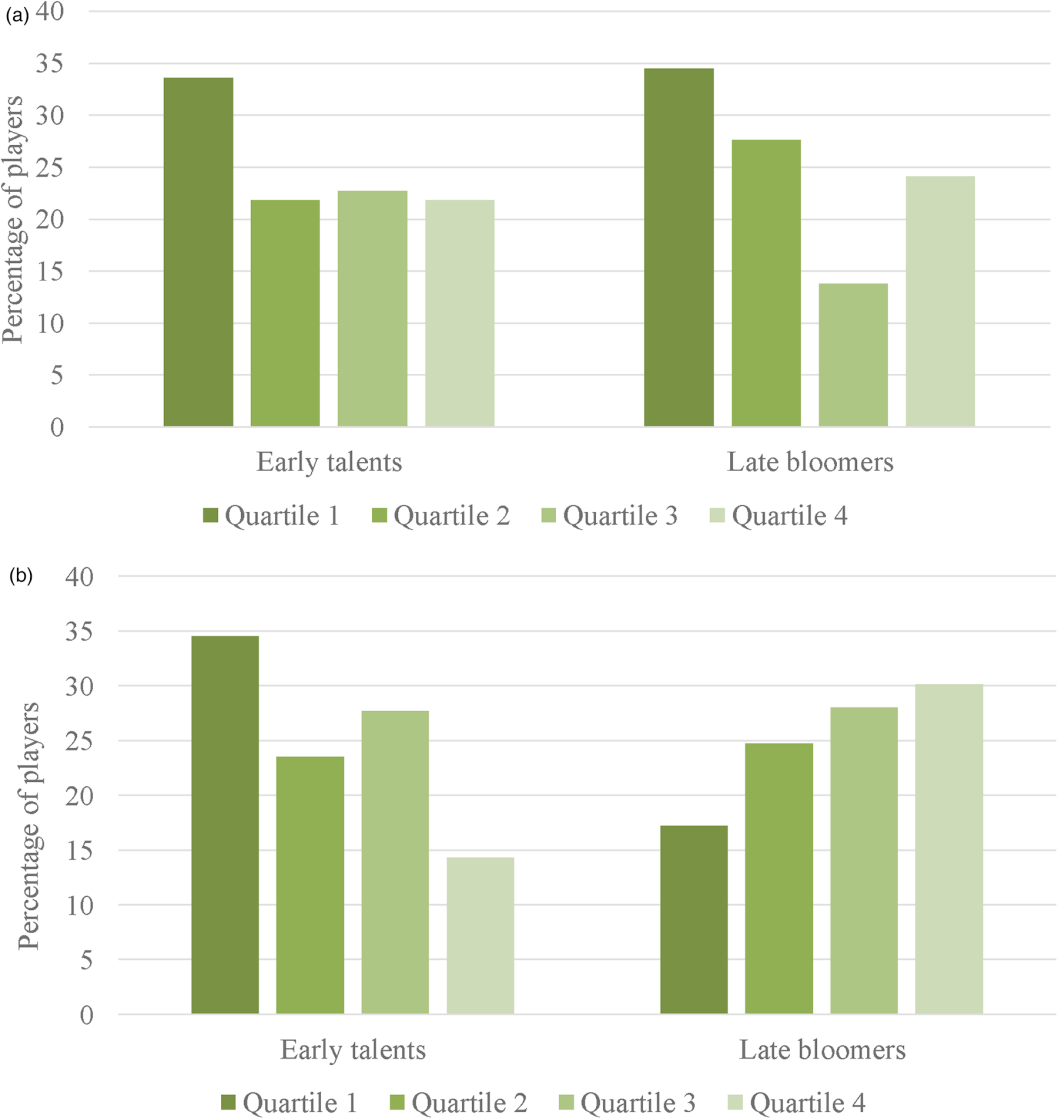
**(a)** Study 1—birth quartile distributions for female early talents and late bloomers. **(b)** Study 1—birth quartile distributions for male early talents and late bloomers.

As can be seen in Figure 1a, an expected within year distribution was revealed on a descriptive level for female early talents and late bloomers. However, these effects were not significantly different from an equal distribution. For the early talents, a small effect size was found, *χ*²(3, *n* = 110) = 4.40, *p* = .22, *w* = .20, *90% CI* [.00, .32], while for the late bloomers a medium sized effect was noted, *χ*²(3, *n* = 29) = 2.59, *p* = .46, *w* = .30, *90% CI* [.00, .51]. As can be seen in Table 2, these results appear to be driven by the third league, because of the number of players per league although, unfortunately, the small cell sizes do not allow for differentiation between leagues. No significant differences were revealed when comparing the distributions between early talents and late bloomers, *χ*²(3, *n* = 139) = 1.27, *p* = .74, *w* = .10, *90% CI* [.00, .18].

**Table 2:** Study 1—number of players differentiated by sexes, league level and developmental pathway.

Sex	League	Early talents				Late bloomers			
		Q1	Q2	Q3	Q4	Q1	Q2	Q3	Q4
Female	1	3	7	6	3	2	1	2	0
	2	14	4	5	7	5	3	0	4
	3	20	13	14	14	3	4	2	3
	Total	37	24	25	24	10	8	4	7
Male	1	9	9	6	3	5	4	4	6
	2	12	6	13	8	4	8	3	5
	3	20	13	14	6	7	11	19	17
	Total	41	28	33	17	16	23	26	28

For the male players, different effects were revealed. A significant within-year effect was found for the early talents, *χ*²(3, *n* = 119) = 10.18, *p* = .02, *w* = .29, *90% CI* [.09, .42], as shown in Figure 1b, while a reversed distribution was visible for the late bloomers, although this effect did not reach statistical significance against an equal distribution, *χ*²(3, *n* = 93) = 3.56, *p* = .31, *w* = .20, *90% CI* [.00, .32]. However, comparing both birth quartile distributions, late bloomers differed significantly from early talents, *χ*²(3, *n* = 212) = 11.97, *p* < .01, *w* = .24, *90% CI* [.09, .33].

## 5 Study 1—discussion

Previous research on German handball talents at the talent selection camps has repeatedly shown small to medium relative age effects (7–9). As expected based on previous findings, the early talent group demonstrated RAEs with small effect sizes (i.e., birth quartile distribution with over-representation of relatively older athletes) (3, 4). As with previous athlete research, females showed smaller relative age effects than males (3, 14).

Interestingly, different distributions were found when comparing male and female samples of late bloomers. In the male sample, the comparison of late bloomers and early talents was statistically significant, indicating differences between these distributions. Descriptively, this difference appeared to be driven by a reverse relative age distribution in the late bloomers, although this main effect was not significant. For the female late bloomers, a different distribution was observed. There were no differences between these late bloomers in comparison to the early talents. In contrast to the male athletes, there was no evidence of a reversed relative age effect. There are several possible explanations for this, but the small sample size of late bloomers (*n* = 29) may have been a contributing factor. Compared with the male athletes (*n* = 93), reaching the higher tiers for females may be more difficult than for males, if they did not make it into the national talent development system. This suggests there is an infrastructure for male players to succeed as late bloomers that is less effective for female athletes (15, 16). Collectively, these distributions could serve as a starting point to contrast the developmental pathways of athletes in German handball in general and between male and female programs in particular.

While this study suggests some intriguing results, there were limitations, including the small female sample noted above. In addition, it would have been interesting to differentiate between the various league levels, but this was not possible due to the small cell sizes between leagues. Future studies might try to develop similar datasets for other sports. Being able to differentiate between league or expertise levels would be helpful for a better understanding of the role relative age effects in the long-term development of athletes. This knowledge might provide us with ideas regarding how relative age effects can be reduced (17).

Overall, this study provides a good first step toward a deeper understanding of relative age effects as a longitudinal phenomenon. However, even within our first study, the developmental path remains a black box, because we did not control for it. For example, an assumption in relative age effect research is that the developmental environment remains constant over time. We explore this assumption in Study 2.

## 6 Study 2—relative age effects in German youth handball players from the first national talent selection to the junior world championships

The German handball talent development system starts with the first national talent selection camp (16). This group is perceived to include players with the greatest “talent” for future success. Approximately 240 14-year-old female players and 240 15-year-old male players present themselves the first time to the youth national coaches. Of these, around 50 players are chosen by the youth national coaches for a second talent selection, of which approximately 20 are chosen for the youth national team.

These youth national team players then train within the national development system (16). However, to prepare for the international tournaments 2-year groups are combined as one youth national team. The two main international competitions are the Youth World Championships and the Junior World Championships. During this period of approximately 5 years, players not only train in the national development system, they also stay within their clubs. While there is a clear reduction of numbers from the talent selection camps to the youth national team, the number of players remains the same across the international tournaments. However, the team's make-up could still change. Some may remain for the whole period, while others might lose their spot, drop-out from the sport, or move up to the team, if they show strong performances in the club system.

**Figure 2:**
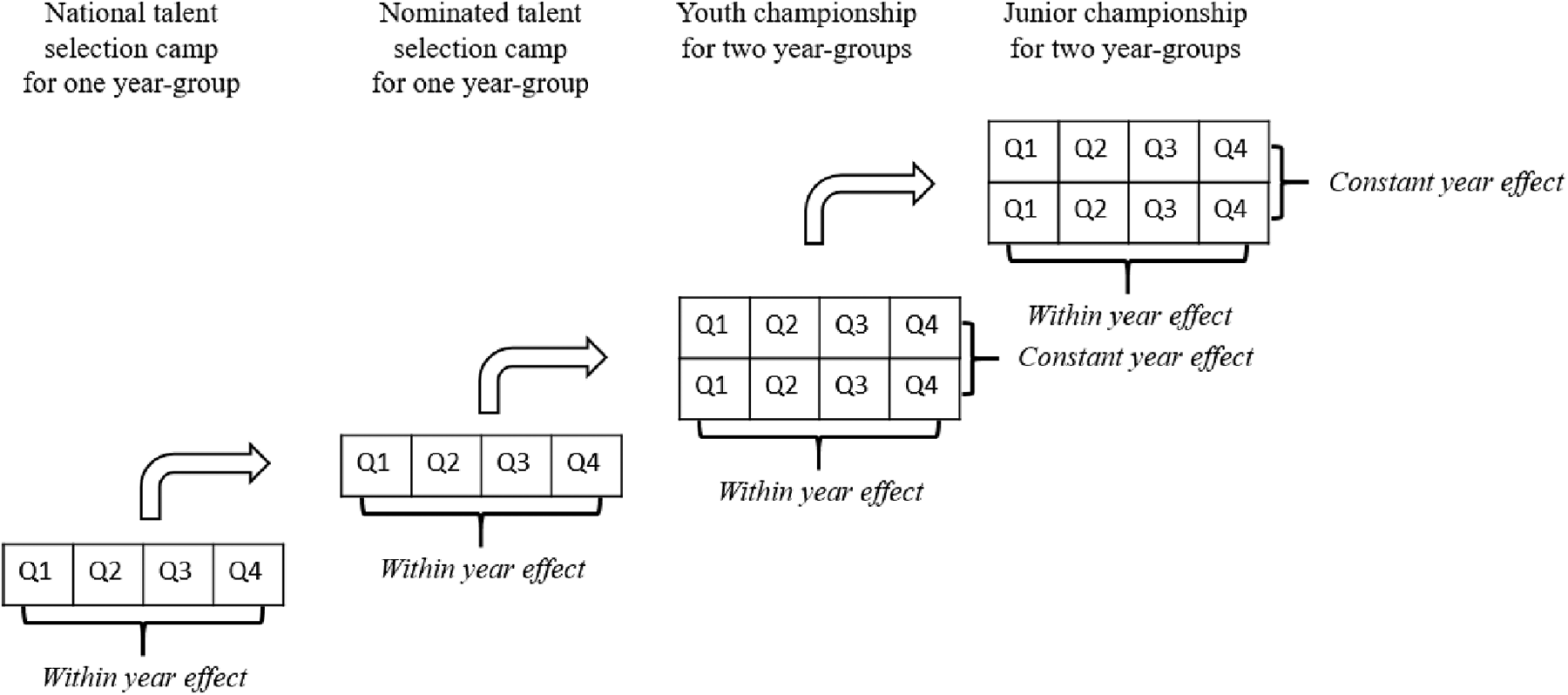
Study 2—talent development pathway in German handball with within year effects and constant year effect.

The complicated structure of the German national talent development system suggests there are two age-groupings at play (cf. Figure 2). While the within-year effect (i.e., the classical RAE) can be investigated during all four points of time, a constant year effect can only be explored during tournament phases, when 2-year bands are combined (9). Because the emphasis is typically on international competition, throughout the tournament phase, younger players in this 2-year band have to compete with older players for spots on the roster. These shifting and competing effects highlight the complexity of age-related effects across athlete development. While some previous research has explored the longitudinal nature of with-in year effects [Study 1 as well as (6)], to the best of our knowledge, changes in constant year effects across development have not been examined.

While most studies look only at the beginning and/or the end of this process, the aim of this study was to consider two relative age effects at different points of time during the national talent development period within the German handball federation. In a first step, we report within-year effects during the talent selection period, because only 1 year band is considered here. As with previous research, we expected small to medium effect sizes for the handball talents (7, 10), and smaller effects for the girls as for the boys (14). For tournament phases, we examined within-year effects and constant-year effects among the athletes who remained in the program compared to those had to drop-out or joined at a later age.

### 6.1 Study 2—methods

#### 6.1.1 Samples

For the present study, birthdates from two samples of young players were taken. First, the German Handball federation provided the birthdates of male athletes born between 1992 and 1997 from their talent development program. Additionally, they provided the data for females in the talent development program born between 1996 and 1999. This information included each athlete's birth date as well as their participation at the first and second talent selection camp, the World youth and World junior championships. These are the four main events during their national talent development career with the German Handball Federation. From the first talent selection camp with 240 participants per birth year, the number of players is reduced to approximately 40 for the second one. For both tournaments, which are played with double year bands, the number of players is reduced to 20 for both birth years together. These are then the players nominated to compete at these international tournaments plus their reserves.

#### 6.1.2 Statistical analyses

Given that the cut-off date in handball is January 1st, players' birth months were re-coded to reflect his or her birth quartile (January–March = quartile 1, April–June = quartile 2, July–September = quartile 3 and October–December = quartile 4) as in study 1. Additionally, we differentiated between athletes in older and younger year groups to test for between-year effects because handball tournaments are played in teams of 2-year bands. As in Study 1 and in line with previous research on birthdate distribution in Germany (8), statistical analyses were conducted against an equal distribution of births across the year. Chi-square tests were calculated to test for within-year and between-year effects. All analyses were conducted with SPSS 29.0. Effect sizes were determined using the software G*power (13). For each effect size, the 90% confidence interval was calculated based on the noncentral Chi-square files provided online by Michael Smithson (http://www.michaelsmithson.online/stats/CIstuff/CI.html).

### 6.2 Study 2—results

*Males*: For the first talent selection, significant within-year effects were revealed for the males, *χ*²(3, *n* = 1282) = 164.18, *p* < .001, *w* = 0.36, *90% CI* [0.31, 0.40]. As expected, the first quartile (37.44%) was over-represented in comparison to the second (27.30%), third (22.70%) and fourth quartiles (12.56%). At the second talent selection camp, a significant within-year effect was observed, *χ*²(3, *n* = 279) = 64.53, *p* < .001, *w* = 0.48, *90% CI* [0.37, 0.57]. Again, the first quartile (43.73%) was over-represented compared to the second (25.45%), third (20.07%) and fourth quartiles (10.75%).

**Figure 3:**
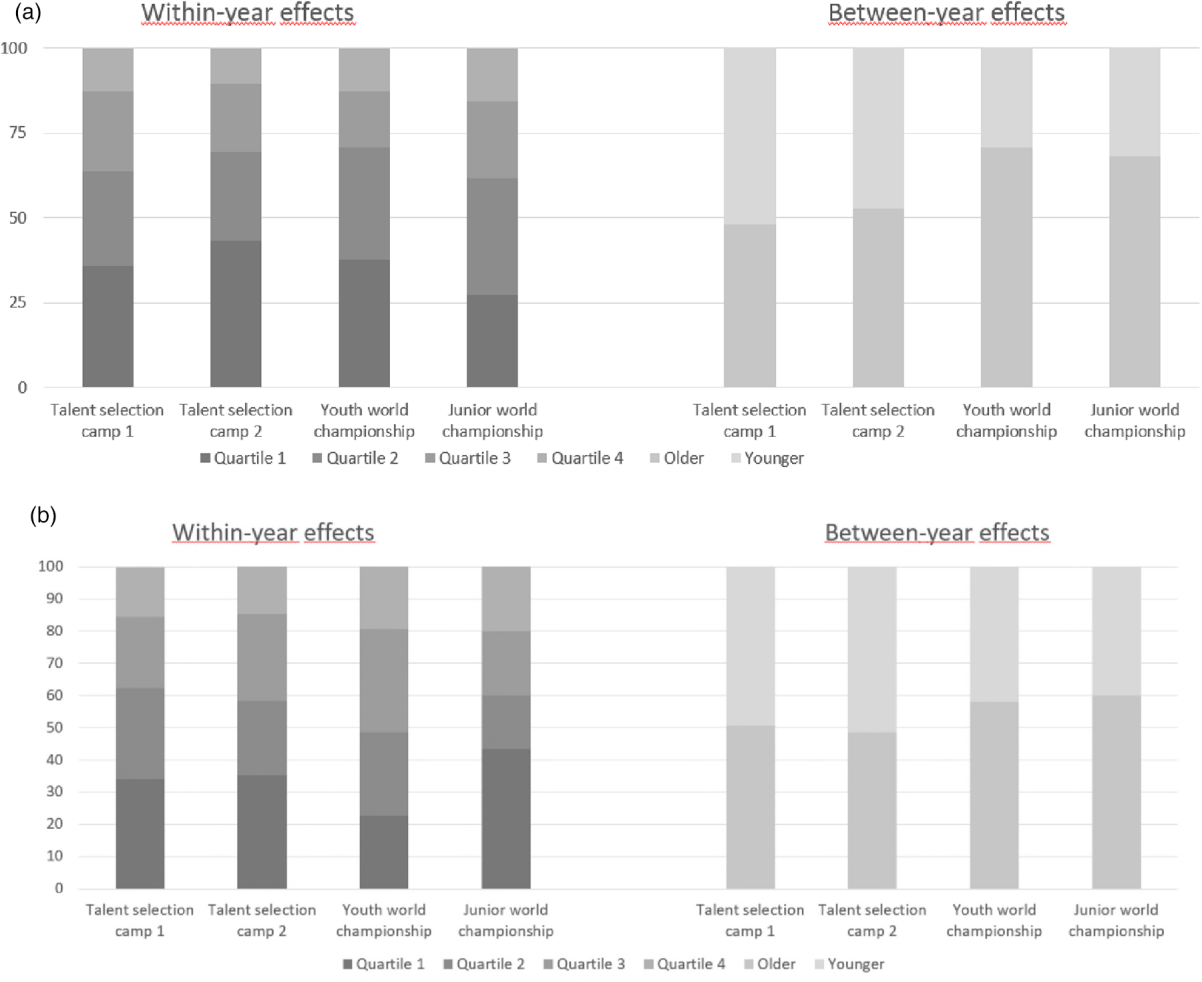
**(a)** Study 2—birth quartile distributions for male players during four different points of time during the national team career. **(b)** Study 2—Birth quartile distributions for female players during four different points of time during the national team career.

When focusing on the birth-date distributions at the youth and junior world championships (cf. Table 3; Figure 3a), players who played both tournaments (remainders) showed no significant within-year effects, *χ*²(3, *n* = 22) = 3.09, *p* = .38, *w* = .37, *90% CI*[.00, .63], but there was a significant between-year effect, *χ*²(1, *n* = 22) = 6.54, *p* = .01, *w* = .55, *90% CI* [.19, .90]. For those who played the youth tournament, but not the later junior one (drop-outs), neither a significant within-year effect, *χ*²(3, *n* = 26) = 6.92, *p* = .07, *w* = .52, *90% CI* [.00, .78], nor a between-year effect was revealed, *χ*²(1, *n* = 26) = 2.46, *p* = .12, *w* = .31, *90% CI* [.00, .63]. Similarly, no effects were found for players who did not play the earlier youth championship—within-year effect, *χ*²(3, *n* = 22) = 2.00, *p* = .57, *w* = .30, *90% CI* [.00, .53], between-year effect, *χ*²(1, *n* = 22) = 0.73, *p* = .39, *w* = .18, *90% CI* [.00, .53].

**Table 3:** Study 2—comparison of birth quartile distributions of drop-outs (i.e., played the youth tournament but not the later junior one), remainders (i.e., played both tournaments) and joiners (i.e., played the junior tournament but not the earlier youth one) between youth and junior world championships of male German handball talents.

Players development	*N*	Q1	Q2	Q3	Q4	Older	Younger
Drop-outs	26	42.3	30.8	19.2	7.7	65.4	34.6
Remainders	22	31.8	36.4	13.6	18.2	77.3	22.7
Joiners	22	22.7	31.8	31.8	12.6	59.1	40.9

Additionally, the older and younger year groups are presented.

*Females*: For the first talent selection, a significant within-year effect was found for female players, *χ*²(3, *n* = 838) = 56.76, *p* < .001, *w* = 0.26, *90% CI* [0.20, 0.31]. As expected, the first quartile (34.13%) was over-represented in comparison to the second (26.49%), third (23.39%) and fourth quartiles (15.99%). At the second talent selection camp, a significant within-year effects was observed, *χ*²(3, *n* = 279 = 17.24, *p* < .001, *w* = 0.30, *90% CI* [0.16, 0.41]. As expected, the first quartile (35.79%) was over-represented in comparison to the second (23.16%), third (26.32%) and fourth quartiles (14.74%).

When focusing on the birth-date distributions of players at the youth and junior world championships (cf. Table 4; Figure 3b), those who played in both tournaments (remainers) had no significant within-year effects, *χ*²(3, *n* = 11) = 1.00, *p* = .80, *w* = .30, *90% CI* [.00,.54], and no between-year effects, *χ*²(1, *n* = 11) = 0.82, *p* = .37, *w* = .27, *90% CI* [.00, .77]. For those who played in the youth tournament but not the later junior one (drop-outs), neither a significant within-year effect, *χ*²(3, *n* = 20) = 2.80, *p* = .42, *w* = .37, *90% CI* [.00, .64], nor a between-year effect was revealed, *χ*²(1, *n* = 20) = 0.20, *p* = .65, *w* = .10, *90% CI* [.00, .45]. However, for the athletes who played only in the junior world championships, a significant within-year effect was found, *χ*²(3, *n* = 19) = 11.95, *p* < .01, *w* = .79, *90% CI* [.31, 1.11], but no significant between-year effects, *χ*²(1, *n* = 19) = 0.47, *p* = .49, *w* = .16, *90% CI* [.00, .53].

**Table 4:** Study 2—comparison of birth quartile distributions of drop-outs (i.e., played the youth tournament but not the later junior one), remainders (i.e., played both tournaments) and joiners (i.e., played the junior tournament but not the earlier youth one) between youth and junior world championships of female German handball talents.

Players development	*N*	Q1	Q2	Q3	Q4	Older	Younger
Drop-out	20	25.0	20.0	40.0	15.0	55.0	45.0
Remainders	11	18.2	36.4	18.2	27.3	63.6	36.4
Joiners	19	57.9	5.3	21.1	15.8	57.9	42.1

### 6.3 Study 2—discussion

In a first step, we explored within-year effects during two talent selection camps in German handball. In line with previous research, there were significant, generally medium-sized, within-year effects for female and male athletes (7–9). Also, similar to previous research, the effects sizes for the females were smaller than for the males (7–9).

A more differentiated picture arose for the within-year effects during the tournaments. For the male athletes, three distinct birth quartile patterns emerged. Players who dropped out (i.e., only participated in the first tournament) showed a classical within-year effect, while in the joiners (i.e., those who only participated in the second tournament) the middle two quartiles were over-represented. For the athletes who participated in both tournaments, the first two quartiles were over-represented. These differences in birth quartile distributions might be support for the underdog hypothesis proposed by Smith and Weir (18). The main idea of the underdog hypothesis is that players who were initially disadvantaged by their birth quartile, find a way to compete on the older level during their development and have therefore an advantage at later stages of their development.

However, this pattern was not found for the female athletes. In females the first quartile was over-represented in the joiners and the third quartile was largest for the drop-outs. Of the athletes who played in both tournaments (the remainers), the second quartile was the most frequent one. While the male sample provides a clearer picture, the distributions for the females are more difficult to interpret. Importantly, the small sample sizes in the female analyses need to be treated with caution and future research should try to use bigger sample sizes over an even longer period of time (5).

In contrast to the findings for the within-year effects, similar results were demonstrated for female and males for the between-year effects. The strongest between-year effect can be seen for the remainers. Similar to previous findings on within-year effects, here between-years effects were stronger in males than females. A possible explanation might be that, as previously noted, the competition for the spots for the males is higher in German handball than for the females (15, 16). However, as argued for the within-year effects, all these effects should be looked at with caution because of the small sample sizes in these analyses.

## 7 General conclusion

The developmental nature of relative age effects is widely supported (3, 4). However, in the decades since Barnsley et al. (19) very few longitudinal studies have been presented (5, 6). The results of the current studies compliment and extend previous work suggesting the non-linearity of athlete development and the impact of RAEs. In study 2, there is evidence that during national youth development programs, different groups of athletes are entering and exiting the national development system over time. From an athlete development perspective, these changing populations are intriguing, although the mechanisms that promote or constrain these changes are currently unclear. Future studies should try to focus more on these phases of athlete development.

Taken together, these studies suggest relative age researchers should embrace longitudinal designs (5, 6). These types of prospective approaches would allow explorations of the association of other variables with the observed relative age effects. For example, integrating aspects of psychological qualities like motivation with physical elements of growth and maturation to determine how they shift and evolve with relative age across development may provide a clearer picture of this phenomena. Certainly, solutions to this persistent inequality [see Webdale et al. (17)] will only come with more advanced approaches than the descriptive designs used in most work in this area.

## Data Availability

The data analyzed in this study is subject to the following licenses/restrictions: the data is not ours, but from the German Handball federation. Requests to access these datasets should be directed to joerg.schorer@uol.de.

## Footnotes

^1^The SPSS-script provided by Michael Smithson calculates the non-centrality parameter for the non-central distributions under which a particular chi²-value cuts off an area of α% (here: 5%) to the right (*λ*_low_) and left (*λ*_low_). Based on these calculations, the lower (*w*_low_) and upper (*w*_upp_) bound of the confidence interval on Cohen’s *w* are obtained via the formula *w*_low_=sqrt[*λ*_low_/N] and *w*_upp_=sqrt[*λ*_upp_/N], respectively.
